# Semi-automated computed tomography Volumetry can predict hemihepatectomy specimens’ volumes in patients with hepatic malignancy

**DOI:** 10.1186/s12880-019-0309-5

**Published:** 2019-02-26

**Authors:** Philipp Mayer, Martin Grözinger, Theresa Mokry, Peter Schemmer, Nina Waldburger, Hans-Ulrich Kauczor, Miriam Klauss, Christof-Matthias Sommer

**Affiliations:** 1 Department of Diagnostic and Interventional Radiology, University Hospital Heidelberg, Heidelberg, Germany; 2 Department of General and Transplant Surgery, University Hospital Heidelberg, Heidelberg, Germany; 3 Division of Transplant Surgery, Department of Surgery, Medical University of Graz, Graz, Austria; 4 Institute of Pathology, University Hospital Heidelberg, Heidelberg, Germany

**Keywords:** Computed tomography volumetry, Hemihepatectomy, Hepatic malignancy

## Abstract

**Background:**

One of the major causes of perioperative mortality of patients undergoing major hepatic resections is post-hepatectomy liver failure (*PHLF*). For preoperative appraisal of the risk of *PHLF* it is important to accurately predict resectate volume and future liver remnant volume (FLRV). The objective of our study is to prospectively evaluate the accuracy of hemihepatectomy resectate volumes that are determined by computed tomography volumetry (*CTV*) when compared with intraoperatively measured volumes and weights as gold standard in patients undergoing hemihepatectomy.

**Methods:**

Twenty four patients (13 women, 11 men) scheduled for hemihepatectomy due to histologically proven primary or secondary hepatic malignancies were included in our study. CTV was performed using a semi-automated module (*S*, hereinafter) (syngo.CT Liver Analysis VA30, Siemens Healthcare, Germany). Conversion factors between *CT volumes* on the one side and *intraoperative volumes and weights* on the other side were calculated using the method of least squares. Absolute and relative disagreements between *CT volumes* and *intraoperative volumes* were determined.

**Results:**

A conversion factor of c = 0.906 most precisely predicted *intraoperative volumes* of exsanguinated hemihepatectomy specimens from *CT volumes* in all patients with mean absolute and relative disagreements between *CT volumes* and *intraoperative volumes* of 57 ml and 6.3%. The use of operation-specific conversion factors yielded even better results.

**Conclusions:**

CTV performed with *S* accurately predicts *intraoperative volumes* of hemihepatectomy specimens when applying conversion factors which compensate for exsanguination. This allows to precisely estimate the FLRV and thus minimize the risk of *PHLF* in patients undergoing major hepatic resections.

## Background

Major hepatic resections are often necessary to achieve curative resection in patients with extensive liver invasion by primary or secondary malignancy [1]. Patients undergoing a major hepatic resection are at increased risk for peri- and postoperative complications [2]. Among these, one of the major causes of perioperative mortality is post-hepatectomy liver failure (PHLF), defined as the impaired ability of the liver to maintain its synthetic, excretory, and detoxifying functions [1]. Many factors influence the risk of PHLF, including patient-related factors such as diabetes and overweight, the presence of underlying parenchymal liver disease such as steatosis, cirrhosis, and chemotherapy effects [3]. However, the size of the future liver remnant volume (FLRV) is considered to be the most important correctable predictor of PHLF [4]. Smaller FLRVs also increase the risk of postoperative infection [5]. Although there is no uniform agreement on the minimum FLRV, most hepatic surgeons consider a FLRV of 25.0% as sufficient in patients without underlying parenchymal liver disease, and a FLRV of at least 40.0–50.0% in patients with severe parenchymal disease [6, 7]. In Western patients without underlying parenchymal disease, the right liver lobe constitutes about two thirds of the total liver volume [4]. However, Abdalla et al. found great interpatient variability of segmental hepatic volumes determined by computed tomography volumetry (CTV) in 102 patients without liver disease [8]. Therefore, precise measurement of liver volumes plays a critical role in preoperative assessment of patients who are planned for major hepatic resections due to primary hepatic tumors or hepatic metastases [1].

Multiple imaging modalities have been exploited to measure the volume of FLR, including computed tomography (CT), magnetic resonance imaging (MRI), as well as ultrasound [4]. Among these, most authors consider CTV to be the current gold standard [6]. However, several studies have reported a significant inaccuracy rate of preoperatively measured liver volumes by manual, semi-automated or automated methods of CTV [9]. Most of these studies focused on living-related liver transplantation (LRLT) and showed over- or underestimation of actual graft volumes by CTV of up to 53.0% compared with intraoperatively measured volumes and weights [10, 11]. One study compared CTV of hepatectomy specimens with intraoperatively measured volumes in patients who underwent partial liver resection for focal liver lesions, with the result that their semi-automated method of CTV overestimates the specimen volume by about 14.0%, on average [9]. However, in this study the segmentation of the resected hemihepatectomy specimens was performed retrospectively according to the resection border visible on the postoperative MR images [9].

With this background, the objective of our study was to evaluate the accuracy of hemihepatectomy resectate volumes that were determined by CTV using a semi-automated Analysis Module (***S***) (syngo.CT Liver Analysis VA30, Siemens Healthcare, Germany) when compared with intraoperatively measured volumes and weights as gold standard in patients undergoing hemihepatectomy for primary or secondary hepatic malignancy.

## Methods

### Patient characteristics

24 patients (13 women, 11 men; mean age: 63 years ± SD [standard deviation] 11 years; range: 30 to 84 years) scheduled for hemihepatectomy due to histologically proven primary or secondary hepatic malignancies were included in our study. Malignancies were cholangiocarcinoma (CC) in 14 cases, hepatocellular carcinoma (HCC) in 1 case, and metastases in 9 cases (6 from colorectal cancer (CRC), 1 from thyroid cancer (TC), 1 from granulosa cell tumor (GCT), and 1 from malignant melanoma (MM)). Histopathologic analysis of the non-neoplastic tissue parts of the hemihepatectomy specimens showed no evidence of steatosis, portal fibrosis with formation of septa or parenchymal necrosis in 14 patients. The resected non-neoplastic liver parenchyma of the other 10 patients showed evidence of steatosis, portal fibrosis with formation of septa and/or parenchymal necrosis. None of the patients had liver cirrhosis. Patient demographics, performed operations, tumor diameters, and histopathological diagnoses are summarized in Table 1. All patients underwent preoperative CT examination for surgical planning. On average, the preoperative CT scan was performed 33 days prior to the operation (range: 1–226 days). CTV was performed using a semi-automated module (***S***) (syngo.CT Liver Analysis VA30, Siemens Healthcare, Germany) in all patients. Left hemihepatectomy was performed in 6 patients, right hemihepatectomy was performed in 11 patients and extended right hemihepatectomy was performed in 7 patients. The middle hepatic vein (MHV) was resected in 11 patients. Volumes (***intraoperative volume***) and weights (***intraoperative weight***) of the resected hemihepatectomy specimens were determined in the operating theatre.

**Table 1:** Patient demographics, performed operations, tumor diameters, and histopathological diagnoses

operation	ID	Age range	Resection of MHV	Tumor entity	Intrahepatic tumor diameter(s) (CT)	Histopathological diagnosis of resected non-neoplastic liver parenchyma
left hemi-hepatectomy	1	55–59 yrs.	no	CC	1.8 cm	portal fibrosis with formation of septa
	2	80–84 yrs.	no	CC	1.4 cm	extensive parenchymal necrosis
	3	65–69 yrs.	yes	CC	3.2 cm	n.e.
	4	60–64 yrs.	yes	CC	11.0 cm	n.e.
	5	65–69 yrs.	yes	CC	5.0 cm	portal fibrosis with formation of septa
	6	75–79 yrs.	no	CC	1.8 cm	portal fibrosis with formation of septa
right hemi-hepatectomy	7	60–64 yrs.	no	3 metastases (CRC)	2.2 cm to 5.7 cm	n.e.
	8	70–74 yrs.	no	CC	6.4 cm	portal fibrosis with formation of septa
	9	50–54 yrs.	no	6 metastases (CRC)	1.2 cm to 3.3 cm	n.e.
	10	65–69 yrs.	no	5 metastasis (MM)	1.2 cm to 6.0 cm	n.e.
	11	60–64 yrs.	yes	CC	2.2 cm	n.e.
	12	75–79 yrs.	no	1 metastasis (CRC)	5.9 cm	n.e.
	13	45–49 yrs.	no	9 metastases (GCT)	1.4 cm to 3.2 cm	n.e.
	14	65–69 yrs.	no	HCC	7.7 cm	n.e.
	15	60–64 yrs.	no	CC (2 foci)	1.4 cm to 10.1 cm	portal fibrosis with formation of septa
	16	70–74 yrs.	no	CC	1.7 cm	n.e.
	17	50–54 yrs.	no	6 metastases (TC)	1.3 cm to 2.9 cm	hepatic steatosis
extended right hemi-hepatectomy	18	30–34 yrs.	yes	CC	2.9 cm	n.e.
	19	60–64 yrs.	yes	3 metastases (TC)	1.8 cm to 13.8 cm	extensive parenchymal necrosis
	20	60–64 yrs.	yes	CC	11.8 cm	n.e.
	21	70–74 yrs.	yes	3 metastases (CRC)	5.1 cm to 9.5 cm	n.e.
	22	55–59 yrs.	yes	5 metastases (CRC)	3.8 cm to 6.7 cm	n.e.
	23	65–69 yrs.	yes	CC	1.0 cm	extensive parenchymal necrosis
	24	55–59 yrs.	yes	CC	1.5 cm	portal fibrosis with formation of septa

*f* female, *m* male, *yrs.* years, *MHV* middle hepatic vein *CC* cholangiocarcinoma, *GCT* granulosa cell tumor, *HCC* hepatocellular carcinoma, *MM* malignant melanoma, *TC* thyroid carcinoma, *n.e.* no evidence of steatosis, portal fibrosis with formation of septa or parenchymal necrosis

### CT scanning

CT examinations of the abdomen were performed on the following CT scanners: Siemens Definition Flash, Siemens Somatom Definition AS 40, Siemens Sensation 40, and Siemens Emotion 16 (256 rows, 40 rows, 40 rows, and 16 rows, Siemens Healthcare, Germany); Philips Brilliance iCT 256 (256 rows, Philips Healthcare, Hamburg, Germany). The CT scan protocol consisted of, at least, a portal-venous phase. Optionally, non-enhanced, arterial and late phases were performed. Accurate timing of the optional arterial phase was ensured by automated bolus tracking in the suprarenal aorta. The portal-venous phase was obtained with a delay of 60 s. Optionally, with an additional delay of 180 s, a late phase was acquired.

All images were reconstructed using a soft tissue convolution Kernel (either B30f, B30s, I30f, or B41s). Slice thicknesses of the reconstructed images were 2 mm in 1 patient, 3 mm in 16 patients, 4 mm in 1 patient, and 5 mm in 6 patients.

### CT volumetry (CTV)

Axial images of the portal-venous phase were used for CTV, hence obtaining the ***CT volume***. CTV was performed with a semi-automated Analysis Module (***S***) (syngo.CT Liver Analysis VA30, Siemens Healthcare, Germany). The liver outline was segmentated automatically, based on a hierarchical, learning-based approach [12]. After detection of the liver outline, hepatic veins (HV) and the portal vein (PV) were segmented semiautomatically. After setting seed points into the hepatocaval confluence and the main stem of the PV, the system automatically segments the HV and PV. Finally, the transection plane was defined in consensus by a medical student (MG), a radiologist with 12 years of experience in abdominal imaging (MK) and an experienced liver surgeon with 21 years of experience (PS). The volumes of the intrahepatic vessels in the liver area marked for resection were included in the *CT volume*. Surrounding extrahepatic vessels (e.g. extrahepatic PV), extrahepatic bile ducts, and the gallbladder were excluded.

### Measurement of intraoperative weights and volumes

*I****ntraoperative weights*** of hemihepatectomy specimens were measured using an electric table scale (CKW6R55-M, Ohaus Corporation, Pine Brook, NJ USA). ***Intraoperative volumes*** of the resected hemihepatectomy specimens were determined by water displacement based on the principle of Archimedes. A smaller bowl filled to the brim with 25 °C sterile physiologic saline was placed into a sufficiently larger bowl. The specimen was placed into the smaller inner bowl and overflowing saline was collected by the larger bowl. After removing the smaller bowl together with the specimen the overflow was accurately measured to 5 ml using a measuring cup.

Before *intraoperative volume/ weight* measurements the specimens were flushed with physiological saline to remove the blood.

### Comparison of preoperatively defined transection planes with actual transection planes according to the resection border visible on postoperative CT images

In 18 out of 24 patients postoperative CT scans were available. The average time interval between surgery and postoperative CT scans was 55 days (range: 5–272 days). The maximum discrepancies between the preoperatively defined transection planes and the actual transection planes according to the resection border visible on postoperative CT images (taking into account the course of the PV and HV braches close to the resection border) were rated as ≤1.0 cm, 1.1 cm to 2.0 cm, or > 2.0 cm in consensus by two radiologists with 5 and 12 years of experience in abdominal imaging (PM and MK).

### Statistical analysis

Statistical analysis was performed using GraphPad Prism Version 7.03 (GraphPad Software, La Jolla, CA, USA). *CT volume* measurements were compared to *intraoperative weights and volumes*. Correlation coefficients (***r***) were calculated between *CT volumes* on the one side and *intraoperative volumes and weights* on the other side, respectively. Conversion factors (***c***) between *CT volumes* on the one side and *intraoperative volumes and weights* on the other side were calculated using the method of linear least squares. This linear least squares fitting technique is a commonly applied form of linear regression and aims to minimize the sum of squared errors. A general conversion factor was determined for all specimens (***c***_***overall***_) and operation-specific conversion factors were determined for the specific type of operation performed (***c***_***specific***_).

*Differences* (expressed in cm^3^) between *CT volumes* (with and without conversion factors) and *intraoperative volumes* were calculated using the following formula:

$$ Difference= CT\  volume- intraoperative\ volume $$

Mean values for *differences* were calculated for all specimens and separately for the specific type of operation performed.

*Absolute disagreements* (expressed in cm^3^) between *CT volumes* (with and without conversion factors) and *intraoperative volumes* were calculated using the following formulas:

$$ Absolute\ disagreement\ \left( without\ conversion\ factor\right)=\vert CT\  volume- intraoperative\ volume\vert $$

$$ Absolute\ disagreement\ \left( with\ general\ convers\mathrm{i} on\  factor\right)=\vert CT\  volume\ast {c}_{overall}- intraoperative\ volume\vert $$

$$ Absolute\ disagreement\ \left( with\ operation- specific\ conversion\ factor\right)=\vert CT\  volume\ast {c}_{specif\mathrm{i}c}- intraoperative\ volume\vert $$

*Relative disagreements* (expressed in %) between *CT volumes* (with and without conversion factors) and *intraoperative volumes* were calculated similarly as previously described using the following formulas [13]:

$$ Relative\ disagreement\ \left( without\ conversion\ factor\right)=\vert \frac{CT\  volume- intraoperative\ volume}{intraoperative\ volume}\ast 100\vert $$

$$ Relative\ disagreement\ \left( with\ general\ conversion\ factor\right)=\vert \frac{CT\  volume\ast {c}_{overall}- intraoperative\ volume}{intraoperative\ volume}\ast 100\vert $$

$$ Relative\ disagreement\ \left( with\ operation- specific\ c\mathrm{o} nversion\ factor\right)=\vert \frac{CT\  volume\ast {c}_{specific}- intraoperative\ volume}{intraoperative\ volume}\ast 100\vert $$

Mean values for *absolute* and *relative disagreements* were calculated for all specimens and separately for the specific type of operation performed.

Please note that *differences* between *CT volumes* and *intraoperative volumes* can be positive (+) or negative (−) depending on whether the *CT volume* or *intraoperative volume* is bigger. In contrast, *absolute* and *relative disagreements* represent moduli (|x|) of volumes and are always positive.

*CT volumes* and *intraoperative volumes* were compared using a two-tailed Wilcoxon signed-rank test in all specimens (*n* = 24), in left hemihepatectomy specimens (*n* = 6), in right hemihepatectomy specimens (*n* = 11), and in extended right hemihepatectomy specimens (*n* = 7).

Using a two-tailed Mann-Whitney U test, relative disagreements (with and without conversion factors) were compared between a) groups with with slice thickness ≤ 3 mm (*n* = 17) and with slice thickness > 3 mm (*n* = 7), b) between groups with resected (*n* = 11) or non-resected MHV (*n* = 13), as well as c) between groups with (*n* = 10) or without (*n* = 14) evidence of steatosis, portal fibrosis with formation of septa and/or parenchymal necrosis of the resected non-neoplastic liver parenchyma.

*P* values < 0.05 were considered significant.

## Results

### CT volumes

**Fig. 1:**
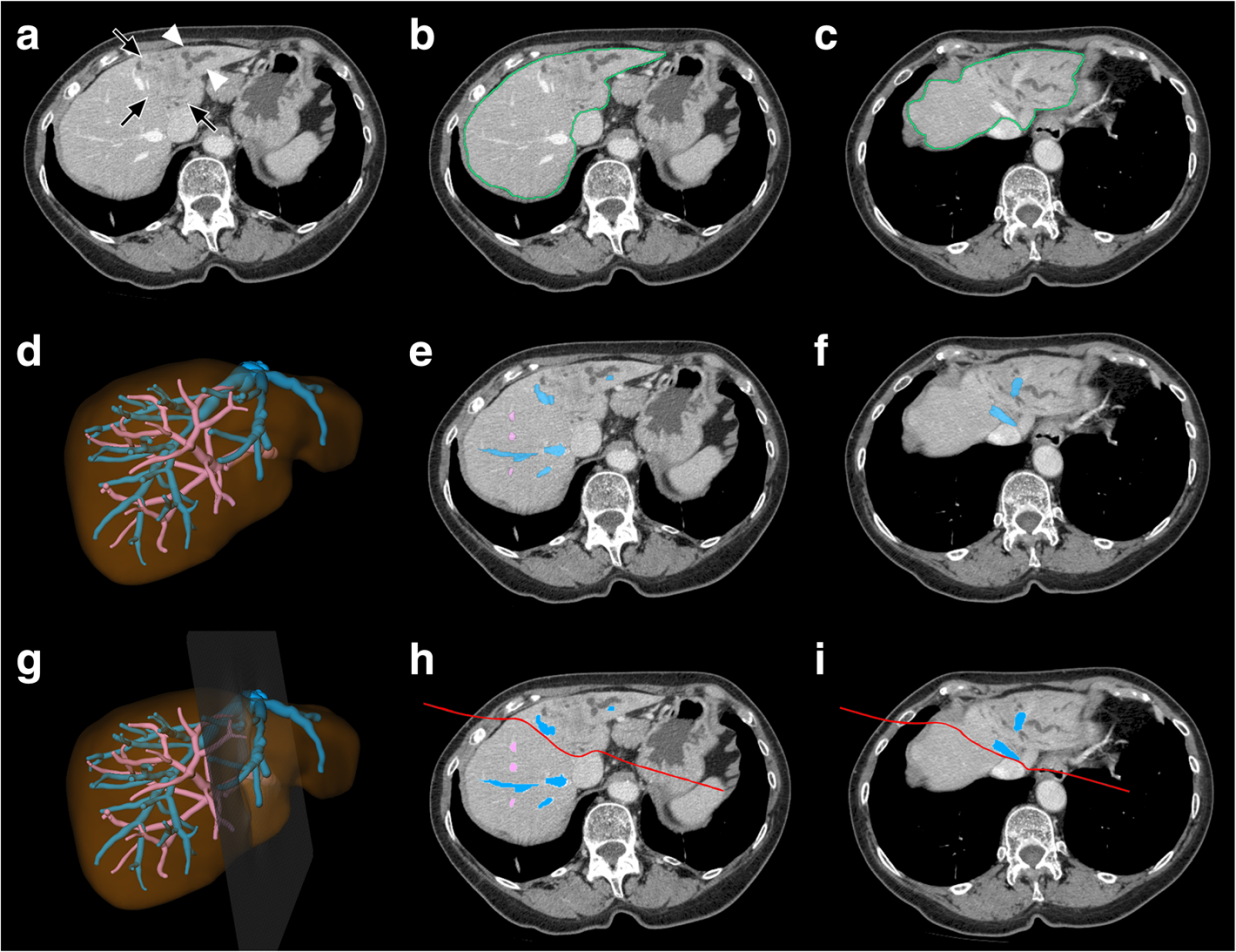
Segmentation process in a patient planned for left hemihepatectomy with resection of the MHV due to intrahepatic CC **a**) Plain axial CT image at the level of the maximum tumor extension shows the hypoattenuated tumor in the atrophic left liver lobe (*black arrows*) with upstream cholestasis (*white arrowheads*). **b**) and **c**) Detection of the liver outline. **d**) – **f**) Detection of the intrahepatic PV (*in pink*) and HV (*in blue*). Note occlusion of the left PV and of branches of the MHV and left HV due to tumor invasion. **g**) – **h**) Definition of the transection plane (*in red*)

**Fig. 2:**
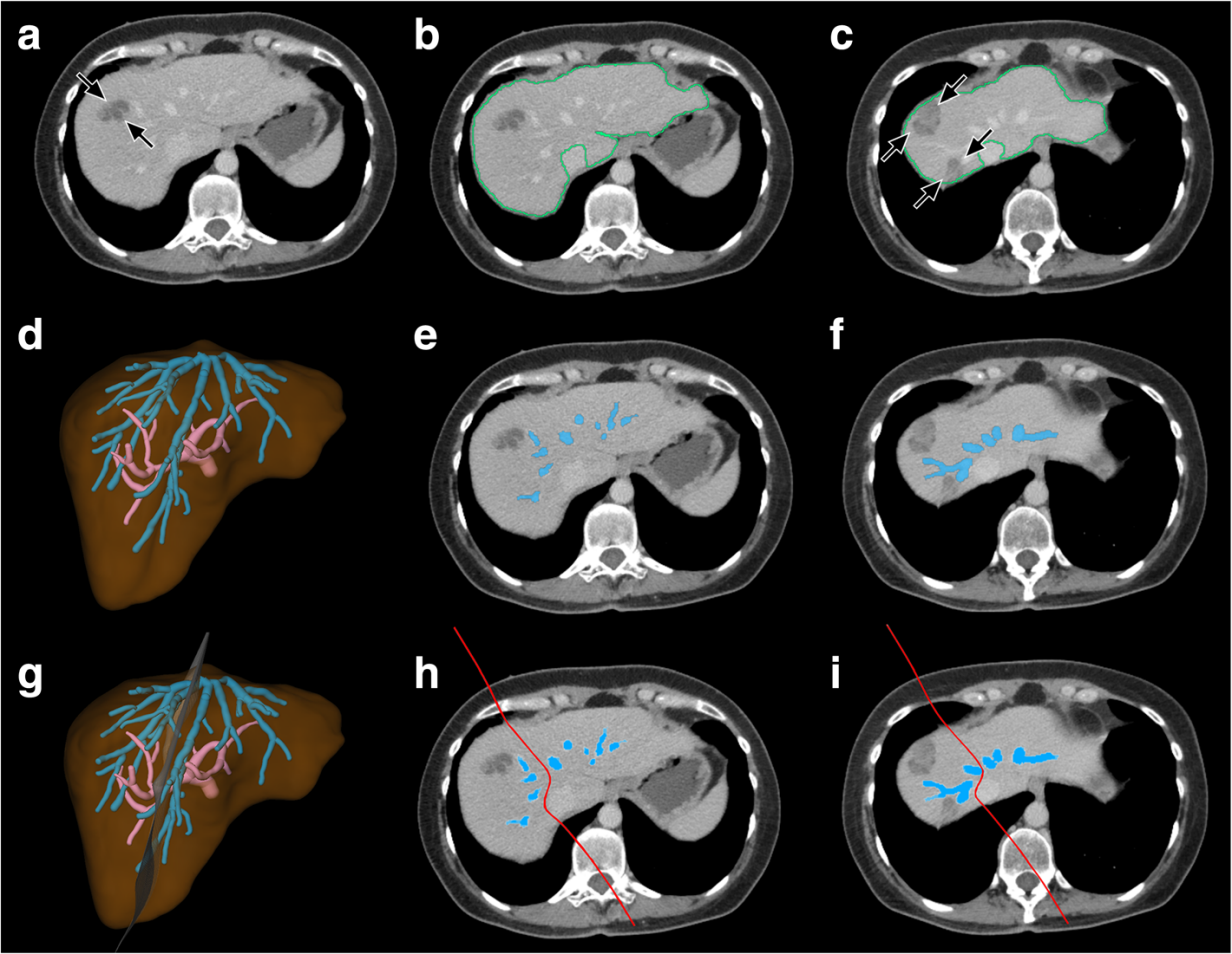
Segmentation process in a patient planned for right hemihepatectomy without resection of the MHV due to metastases of GCT **a**) Plain axial CT image shows a hypoattenuated metastasis in liver segment 8 (*black arrows*). **b**) and **c**) Detection of the liver outline. Further metastases are depicted at the level of the proximal MHV in **c**) (*black arrows*). **d**) – **f**) Detection of the intrahepatic PV (*in pink*) and HV (*in blue*). **g**) – **h**) Definition of the transection plane (*in red*)

**Fig. 3:**
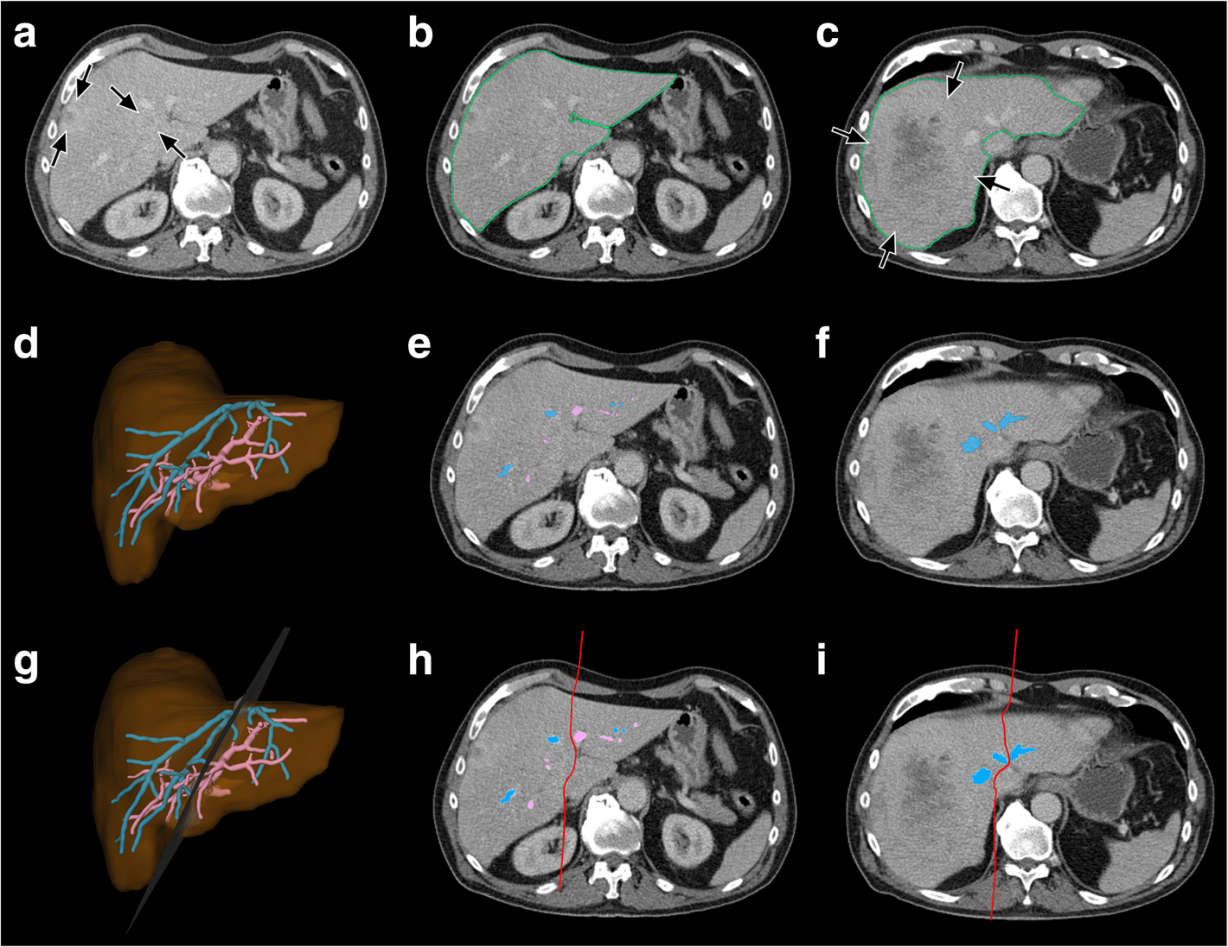
Segmentation process in a patient planned for extended right hemihepatectomy due to metastases of TC **a**) Plain axial CT image shows small peripherally hyperattenuated metastases (*black arrows*). **b**) and **c**) Detection of the liver outline. Another large metastasis is depicted at the level of the proximal MHV in **c**) (*blue arrows*). **d**) – **f**) Detection of the intrahepatic PV (*in blue*) and HV (*in pink*). **g**) – **h**) Definition of the transection plane (*in red*)

The mean *CT volumes* of the hemihepatectomy specimens determined by ***S*** were 1056 cm^3^ overall (range: 138–2245 cm^3^, *n* = 24), 281 cm^3^ in left hemihepatectomy specimens (range: 138–569 cm^3^, *n* = 6), 1070 cm^3^ in right hemihepatectomy specimens (range: 587–1512 cm^3^, *n* = 11), and 1697 cm^3^ in extended right hemihepatectomy specimens (range: 1144–2245 cm^3^, *n* = 7). The *CT volumes* included the volumes of the liver vessels in the analysis areas. The segmentation process for three example patients is shown in Figs. 1, 2, 3.

### Intraoperative volumes and weights

The mean *intraoperative volumes* were 950 cm^3^ overall (range: 125–2010 cm^3^, *n* = 24), 253 cm^3^ in left hemihepatectomy specimens (range: 125–515 cm^3^, *n* = 6), 918 cm^3^ in right hemihepatectomy specimens (range: 475–1310 cm^3^, *n* = 11), and 1598 cm^3^ in extended right hemihepatectomy specimens (range: 1018–2010 cm^3^, *n* = 7).

The mean *intraoperative weights* were 988 g overall (range: 162–2112 g, *n* = 24), 282 g in left hemihepatectomy specimens (range: 162–553 g, *n* = 6), 956 g in right hemihepatectomy specimens (range: 516–1306 g, *n* = 11), and 1642 g in extended right hemihepatectomy specimens (range: 1070–2112 g, *n* = 7).

### Comparative data analysis

The maximum discrepancies between the preoperatively defined transection planes and the actual transection planes according to the resection border visible on postoperative CT images (taking into account the course of the PV and HV braches close to the resection border) were rated as ≤1.0 cm in 11 specimens, as 1.1 to 2.0 cm in 7 specimens, and as > 2.0 cm in 0 specimens.

*CT volumes* determined by ***S*** showed strong significant correlations with *intraoperative volumes and weights* overall (*r* = 0.992, *p* < 0.001, and *r* = 0.987, *p* < 0.001, *n* = 24), in left hemihepatectomy specimens (*r* = 0.989, *p* < 0.001, and r = 0.995, *p* < 0.001, *n* = 6), in right hemihepatectomy specimens (*r* = 0.995, *p* < 0.001, and *r* = 0.991, *p* < 0.001, *n* = 11), and in extended right hemihepatectomy specimens (*r* = 0.968, *p* < 0.001, and *r* = 0.941, *p* = 0.002, *n* = 7).

**Fig. 4:**
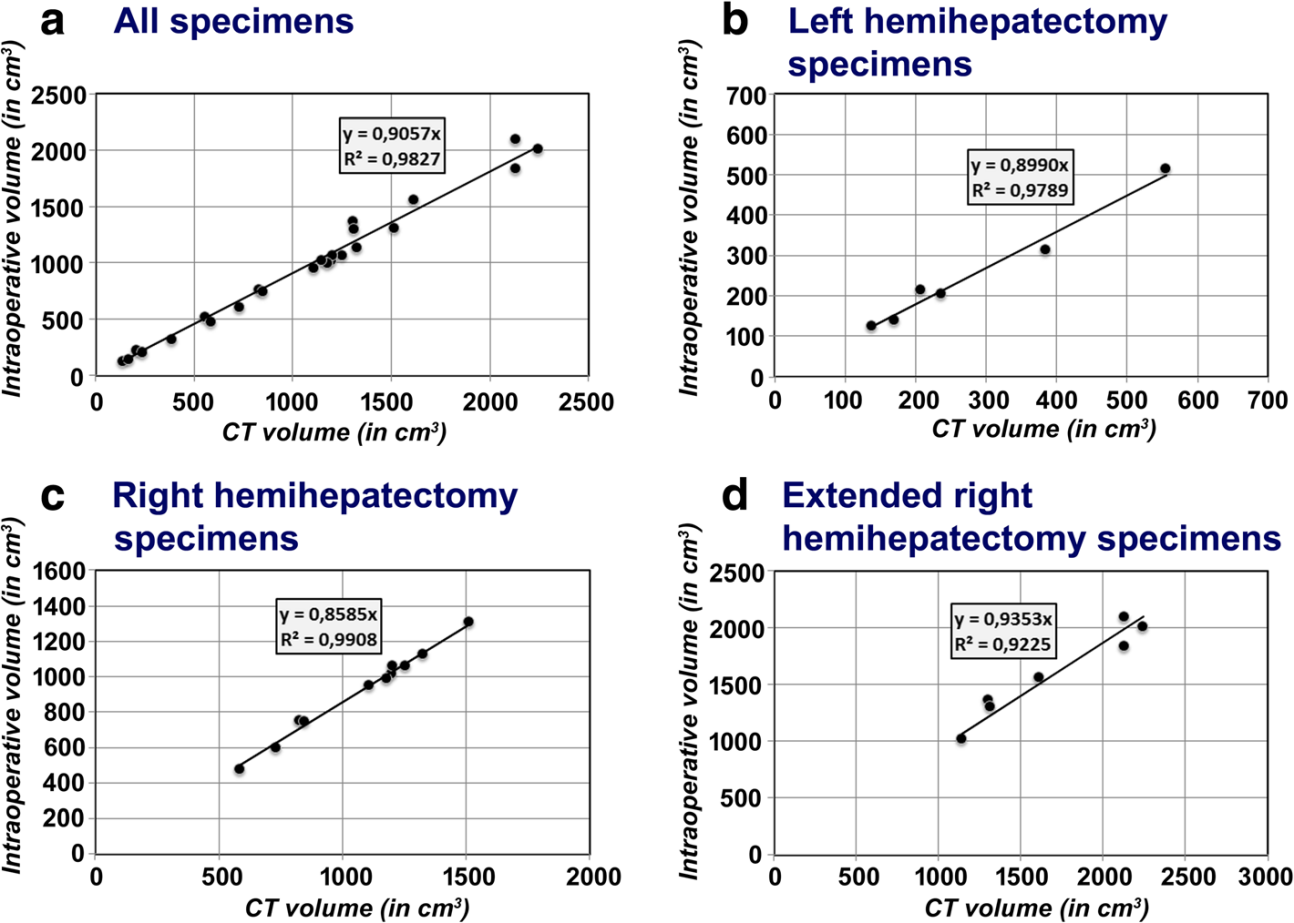
Results of the regression analyses of *intraoperative volumes* and *CT volumes* The linear regression analysis with the method of least-squares was performed for **a**) all specimens, and for **b**) – **d**) each specific type of operation performed

Correlation coefficients and conversion factors calculated by linear regression analyses using the method of least squares are shown in Table 2. Scatter plots with the results of this regression analyses are shown in Fig. 4.

**Table 2:** Correlation coefficients and conversion factors

		Correlation coefficient with *CT volume*	Conversion factor from *CT volume* (in cm^3^)
All patients (*n* = 24)	*Intraoperative volume* (in cm^3^)	0.992*	0.907
	*Intraoperative weight* (in g)	0.987*	0.931
Patients with left hemiheptectomy (*n* = 6)	*Intraoperative volume* (in cm^3^)	0.989*	0.899
	*Intraoperative weight* (in g)	0.995*	0.995
Patients with right hemihepatectomy (*n* = 11)	*Intraoperative volume* (in cm^3^)	0.995*	0.860
	*Intraoperative weight* (in g)	0.991*	0.892
Patients with extended right hemihepatectomy (*n* = 7)	*Intraoperative volume* (in cm^3^)	0.968*	0.935
	*Intraoperative weight* (in g)	0.941*	0.954

Correlation coefficients that are statistically significant are marked with*

Average *intraoperative volumes and weights* (in cm^3^ and g) were smaller than *CT volumes* (in cm^3^), except for *intraoperative weights* (in g) compared to *CT volumes* (in cm^3^) in left hemihepatectomy specimens. *Differences* between mean *CT volumes* and mean *intraoperative volumes* were 106 cm^3^ overall (*n* = 24), 29 cm^3^ in left hemihepatectomy specimens (*n* = 6), 152 cm^3^ in right hemihepatectomy specimens (*n* = 11), and 99 cm^3^ in extended right hemihepatectomy specimens (*n* = 7).

*Differences* between mean *CT volumes* and mean *intraoperative volumes* as well as mean *absolute* and *relative disagreements* between *CT volumes* (with and without conversion factors) and *intraoperative volumes* are shown in Table 3. CT volume was bigger than *intraoperative volume* in all right hemihepatectomy specimens. Thus, the *absolute disagreement* was identical to the *difference* between mean *CT volumes* and mean *intraoperative volumes* in this specimen group. However, *CT volumes* were smaller than *intraoperative volumes* in one extended right hemihepatectomy specimen and in one left hemihepatectomy specimen. Therefore, *absolute disagreements* and *differences* between mean *CT volumes* and mean *intraoperative volumes* were not identical in these specimen groups.

**Table 3:** Differences between mean CT volumes and mean intraoperative volumes as well as mean absolute and relative disagreements between CT volumes and intraoperative volumes

	*Differences* between mean *CT volumes* and mean *intraoperative volumes*	Mean *absolute* and *relative disagreements* of *CT volumes* (with and without conversion factors) and *intraoperative volumes*		
		without conversion factor	with correction by general conversion factor	with correction by operation-specific conversion factor
All patients (*n* = 24)	106 cm^3^*	112 cm^3^/13.1%	57 cm^3^/6.3%	41 cm^3^/4.6%
Patients with left hemihept-ectomy (*n* = 6)	29 cm^3^	32 cm^3^/13.2%	16 cm^3^/6.6%	16 cm^3^/6.5%
Patients with right hemihepat-ectomy (*n* = 11)	152 cm^3^*	152 cm^3^/16.9%	52 cm^3^/6.0%	19 cm^3^/2.5%
Patients with extended right hemihepat-ectomy (*n* = 7)	99 cm^3^	116 cm^3^/7.2%	99 cm^3^/6.3%	95 cm^3^/6.1%

Differences between CT volumes and intraoperative volumes that are statistically significant (using a two-tailed Wilcoxon signed-rank test) are marked with*

Using a two-tailed Wilcoxon signed-rank test, *differences* between *CT volumes* and *intraoperative volumes* were statistically significant when calculated for all specimens (*p* < 0.001, *n* = 24) and for right hemihepatectomy specimens (*p* = 0.001, *n* = 11), but not statistically significant when calculated for left hemihepatectomy specimens (*p* = 0.063, *n* = 6) or for extended right hemihepatectomy specimens (*p* = 0.109, *n* = 7). *CT volume* overestimated the hemihepatectomy specimens’ volume with an average of 11.1% when compared to *intraoperative volume* in all specimens (n = 24).

Using a two-tailed Mann-Whitney U test, mean *relative disagreements* (with and without conversion factors) were not statistically different between a) groups with slice thicknesses ≤3 mm (*n* = 17) and > 3 mm (*n* = 7), b) between groups with resected (*n* = 11) and non-resected MHV (*n* = 13), as well as c) between groups with (*n* = 10) and without evidence of steatosis, siderosis, fibrosis, and/or inflammatory infiltration of the resected non-neoplastic liver parenchyma (*n* = 14). The exact *p* values are summarized in Table 4.

**Table 4:** Comparisons of relative disagreements of CT volumes and mean intraoperative volumes in different patient groups

	Without conversion factor	With general conversion factor c_overall_	With operation-specific conversion factor c_specific_
Slice thickness ≤ 3 mm (*n* = 17) vs. > 3 mm (*n* = 7)	*p* = 0.569	*p* = 0.897	*p* = 0.631
With (*n* = 11) vs. without resection of the MHV (*n* = 13)	*p* = 0.374	*p* = 0.865	*p* = 0.082
With (*n* = 10) vs. without without evidence of steatosis, siderosis, fibrosis, and/or inflammatory infiltration of the resected non-neoplastic liver parenchyma (*n* = 14)	*p* = 0.772	*p* = 0.219	*p* = 0.466

Using a two-tailed Mann-Whitney U test, relative disagreements are not statistically different between groups with different slice thicknesses, between groups with vs. without resection of the middle hepatic vein (MHV), or between groups with vs. without evidence of parenchymal damage

## Discussion

Over the past years, liver surgery has become more aggressive in oncological patients with the objective to achieve microscopically radical resection margins and, hence, better survival [6]. One of the major factors which influences procedural success is the size of the future liver remnant (FLR) [7]. The FLR is used as a surrogate for the risk of postoperative liver failure [14]. A smaller FLR is associated with an increased risk of postoperative infection and severe hepatic dysfunction [5]. Imaging-based volumetry is being utilised in clinical practice with increasing frequency to predict the size of the FLR [7]. CT volumetry (CTV) is often considered the current gold standard [6]. However, several studies have reported a significant inaccuracy rate of preoperatively measured liver volumes by CTV [9], which makes it difficult to preoperatively estimate the risk of perioperative morbidity and mortality in patients who are planned for major hepatic resections.

In this context, the objective of our study was to evaluate the accuracy of hemi-hepatectomy resectate volumes, determined by CTV using a semi-automated segmentation software (***S***) when compared with *intraoperative volumes and weights* in patients undergoing hemihepatectomy for primary or secondary hepatic malignancy. 24 hemihepatectomy specimens were evaluated prospectively. Similarly to most previous studies in the field of CTV of the liver, the volumes of the intrahepatic vessels of the hemihepatectomy specimens were included in, and the volumes of major extrahepatic vessels were excluded from, the *CT volumes* and *intraoperative volumes and weights*, respectively [11, 15, 16].

We have shown a good correlation between *CT volumes* based on venous CT-phase on the one side, and *intraoperative volumes and weights* on the other side. However, *CT volume* overestimated the hemihepatectomy specimens’ volume by an average of 11.1% when compared to *intraoperative volume* in all specimens when no conversion factor was applied. Average *intraoperative weights* (in g) were also smaller than *CT volumes* (in cm^3^), except in left hemihepatectomy specimens. This exception could be attributable to measuring inaccuracies in the comparatively small left hemihepatectomy specimens. Mean relative disagreements of *CT volumes* (with and without conversion factors) and *intraoperative volumes* were not statistically significant in patients with or without evidence of steatosis, portal fibrosis with formation of septa and/or parenchymal necrosis of the resected non-neoplastic liver parenchyma.

The overestimation of liver volumes by CTV is in line with the results of several previous studies [11, 15–17]. A majority of these studies focused on CTV in the field of LRLT [7]. In a study by Schroeder et al., a manual method of CTV overestimated graft-volumes in 10 of 13 donors in LRLT [17]. But, it is noteworthy that graft-volume was determined by actual weighing and assuming a 1:1 conversion factor from grams to cm^3^ [17]. Radtke et al. reported a 20.9% mean overestimation error for graft-volumes by CTV, based on venous CT-phase using a semi-automatic modified live-wire algorithm in 43 adult donors in LRLT [16]. Preoperative *CT volumes* of liver specimens were reported to be 14.0% larger on average than *intraoperatively measured volumes* in a study by Karlo et al. of patients undergoing partial liver resection [9]. According to the work of Hwang et al. and Niehues et al., blood perfusion is the most relevant factor that is accountable for the systematic differences between in vivo CTV and ex vivo water displacement volumetry [15, 18]. Hwang et al. demonstrated a much smaller *difference* between blood-filled graft volumes and volumetric graft volumes than between blood-free graft weight and volumetric graft volumes in 12 right lobe grafts in LRLT, and they proposed a conversion factor of 1.220 between blood-free graft weight and blood-filled graft volume [18]. Niehues et al. concluded that blood perfusion was the only relevant factor leading to their 13.0% systematic *difference* between whole liver volumes determined by CTV and liver volumes measured by water displacement volumetry in their pig animal model [15]. Our results support the use of conversion factors to predict the actual weight of liver specimens more reliably, on the basis of CTV and we approve that blood filling of the specimens seems to account for much of the *differences* between *CT volumes* and *intraoperatively measured volumes*.

In our study, a conversion factor of c = 0.906 most precisely predicted *intraoperative volumes* of exsanguinated hemihepatectomy specimens from *CT volumes* in all patients with mean absolute and relative disagreements between *CT volumes* and *intraoperative volumes* of 57 ml and 6.3%. The use of operation-specific conversion factors, i.e. specific for left hemihepatectomy, right hemihepatectomy, or extended right hemihepatectomy specimens, yielded even better results. The different *disagreements* regarding *CT volumes* and *intraoperative volumes* in the different operation-groups could be due to differing degrees of exsanguination of the specimens.

A number of other factors are known to influence CTV of liver specimens, among them being technical factors of the examination technique and image reconstruction (e.g. contrast agent phase in multiphasic CT imaging, the slice thickness), the method of segmentation (manual, semi-automated, automated) and patient-related factors (nutrition, circadian variations, physical activity) [16, 19, 20].

The method of CTV influences accuracy, precision and rapidity [7]. The manual method of CTV of the liver was initially described by Heymsfield et al. as manually tracing the liver contour in each CT slice image and then multiplying the area by slice thickness [21]. The biggest benefit of semi-automated and automated methods of CTV is an increase of rapidity when comparing automated methods to semi-automated methods, and when comparing semi-automated methods to manual techniques [7, 22]. However, to date automated methods of CTV often provide suboptimal results in CT images that have missing edges due to similar density of adjacent tissues or organs, and in most cases semi-automated methods of CTV are reported to outperform automated methods [7]. Our study could show a good performance of the semi-automated software ***S***. Ling et al. reported that, when compared to other semi-automated and automated liver segmentation approaches, the precision of ***S*** is at the upper end of the scale [12]. However, we have not compared the performance of ***S*** directly to other methods of CTV.

Radtke et al. investigated the impact of the contrast agent phase in multiphasic CT imaging in the field of CTV [16]. They found that the native phase provided mean graft-volumes that were smaller compared to mean graft-volumes measured by CTV with venous phase, and that CTV with native phase overestimated actual graft-volumes to a lesser degree [16]. They hypothesized that the difference might be attributable to osmotic effects which increase hepatic intravascular water content following the intravenous infusion of the contrast agent [16], although we consider partial volume effects to be more likely. Similar to our approach with CTV, the volumes of intrahepatic vessels were included in their graft-volumes determined by CTV, and major extrahepatic vessels were excluded [16]. However, the venous phase is typically preferred for CTV by most authors because it delineates the vascular anatomy better than unenhanced CT [7]. Since exact volume estimation by CTV relies on precise segmentation it is intuitive that one can expect more accurate results utilizing CT images with smaller slice thickness [7]. It was reported that liver volumes determined by CTV increase with smaller slice thickness [19]. This was attributed to reduced errors from partial volume effects with thinner CT image slices [19]. The disadvantage of using thinner CT image slices is the increased expenditure of time, especially for manual methods of CTV [23]. Hori et al. concluded that 5-mm-thick CT image slices are sufficient for CTV of liver grafts in LRLT if a maximum error of 5.0% in the calculated volume is acceptable [19]. In a study by Reiner et al., MDCT images of the abdomen were reconstructed using the slice thicknesses 2, 4, 6, and 8 mm, and total liver volumes were measured using a semi-automated method of CTV [23]. A statistical difference was seen only between volumes based on 2-mm versus 8-mm slices [23]. However, the semi-automated segmentation module ***S*** had been tested on a highly heterogenous dataset containing 75 volumes of 6 different organs and was reported to be robust against variations of slice thickness, scanning protocols and contrast agent phase [12]. In line with this, the accuracy of *CT volumes* in datasets with low slice thickness (≤ 3 mm) was not statistically different from the accuracy of *CT volumes* in datasets with high slice thickness (> 3 mm) when compared to *intraoperative volumes*.

The discrepancies between the preoperatively defined transection planes and the actual transection planes according to the resection border visible on postoperative CT images were comparatively low. An explanation for this could be that the transection planes on the preoperative CT scans were defined in concordance with the liver surgeon who also operated on the patients.

There were limitations to our study. First, we quite certain cannot exclude that the heterogeneity regarding CT scanners, reconstruction kernels and slice thickness of reconstructed images represent confounders in our study, but ***S*** was shown to be robust in a highly heterogeneous dataset. Second, the absolute time to perform CTV using ***S*** was not recorded systematically and the performance of ***S*** was not compared directly to a manual method of CTV. Third, we did not include data on patient nutrition, physical activity, and times of day in our statistical analysis, although liver volume was reported to be dependent on these variables [20].

## Conclusion

CTV performed with ***S*** yielded good measurements of *CT volumes* of hemihepatectomy specimens when compared to actual specimens’ volumes and weights. The difference can be explained by the fact that blood filling of the specimens was included in the CT volumes performed with ***S***, but it was excluded in the intraoperatively measured volumes. Therefore, we propose the use of conversion factors which allow to predict *intraoperative volume and weights* from CT scans of hemihepatectomy specimens more precisely. This allows to precisely estimate the volume of a future liver remnant and thus minimize the risk of *PHLF* in patients undergoing major hepatic resections.

## Abbreviations

c: Conversion factor
CC: Cholangiocarcinoma
c_overall_: General conversion factor for all specimens
c_specific_: Operation-specific conversion factor
CT: Computed tomography
CTV: Computed tomography volumetry
f: Female
FLR: Future liver remnant
FLRV: Future liver remnant volume
GCT: Granulosa cell tumor
HV: Hepatic vein(s)
LRLT: Living-related liver transplantation
m: Male
MHV: Middle hepatic vein
MM: Malignant melanoma
MRI: Magnetic resonance imaging
n.e.: No evidence of steatosis, portal fibrosis with formation of septa or parenchymal necrosis
PHLF: Post-hepatectomy liver failure
PV: Portal vein
*S*: Syngo.CT Liver Analysis VA30
TC: Thyroid cancer
